# Hypothermia ameliorates blast-related lifespan reduction of *C. elegans*

**DOI:** 10.1038/s41598-018-28910-z

**Published:** 2018-07-12

**Authors:** Nicholas B. Angstman, Hans-Georg Frank, Christoph Schmitz

**Affiliations:** Chair of Neuroanatomy, Institute of Anatomy, Faculty of Medicine, LMU Munich, Munich, Germany

## Abstract

Blast-related mild traumatic brain injury induces significant long-term health issues, yet treatment procedures remain underdeveloped. Therapeutic hypothermia has been postulated as a potentially effective therapy. In a *Caenorhabditis elegans* model, we demonstrate a dose-dependent reduction in lifespan following exposure to blast-like shock waves. Using polyvinyl alcohol, we show that cavitation is a key injurious factor in the damaging shock wave component. Short and long lifespan *C. elegans* mutants demonstrated the interaction of genetic and environmental longevity-determining factors. Hypothermia reduced the long term effect of shock wave exposure. Thus, we present an effective *C. elegans* model of long term effects of blast-related mild traumatic brain injury, as well as evidence of the merit of therapeutic hypothermia as a therapy option following blast exposure.

## Introduction

Blast-related mild traumatic brain injury (br-mTBI) has been shown to induce long-term effects including, but not limited to, post-traumatic stress disorder and depression^1^, and reduced life expectancy^2^. While the effects of br-mTBI have been well described, there lacks a deeper understanding of the connecting steps between a damage causing event and the development of disease. Due to delayed onset of disease, as well as complexity in studying the nervous system of mammals, connection between cause and known damaging effects is not yet completely understood in br-mTBI. While mammalian models are an effective and commonly used tool to investigate br-mTBI, limitations such as low sample size, length of life, and complexity highlight the need for other effective models of br-mTBI^3^. Despite such shortcomings, progress has still been made in the investigation of potentially effective therapies following traumatic brain injury. One example, therapeutic hypothermia, has previously shown promise in recovery following moderate to severe traumatic brain injury (reviewed in^4^), although results appear mixed. A recent randomized controlled trial found that hypothermia treatment did not improve outcomes in patients with intracranial hypertension after traumatic brain injury^5^. Due to the limiting factors in the usage of mammalian models, therapeutic hypothermia has yet to be investigated for br-mTBI, despite suggestion that it may be an attractive therapeutic intervention to prevent reduced lifespan following br-mTBI^6–8^.

We have previously shown that *C. elegans* offer a viable alternative to mammalian models, as exposure to shock waves results in behavioral changes that share key features with br-mTBI in humans: initial loss of consciousness followed by recovery^9^. Furthermore, we showed that cavitation was an important factor in causing the observed behavioral change. In the present study, we hypothesized that (i) exposure to shock waves reduces *C. elegans* lifespan in the long term, (ii) this effect is partially dependent on the presence of cavitation, and (iii) therapeutic hypothermia improves the long term outlook of *C. elegans* survival.

## Results and Discussion

**Figure 1:**
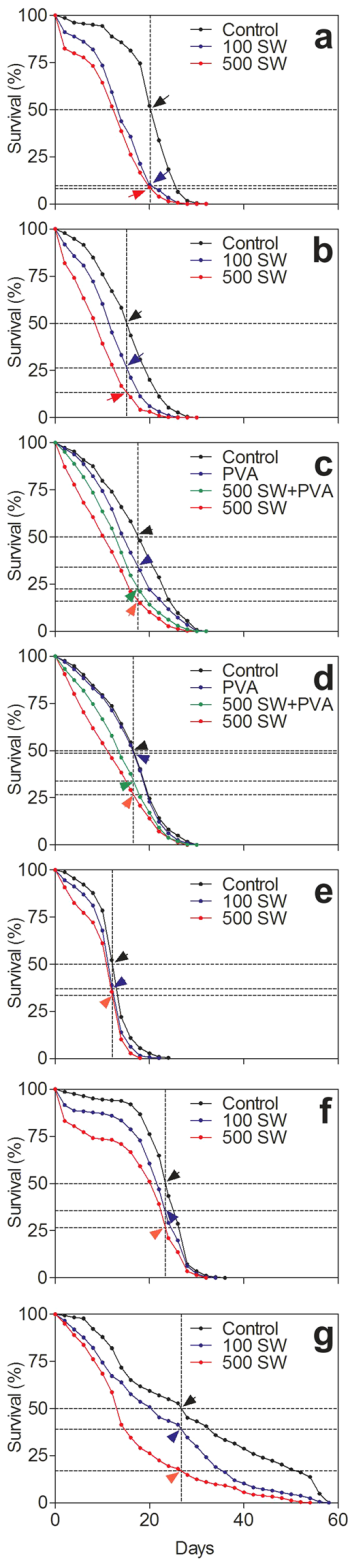
Shock wave exposure decreases lifespan. (**a**) Lifespans of N2 *C. elegans* raised in liquid cultures following shock wave exposure. (**b**) Lifespans of N2 worms raised on NGM agar plates following shock wave exposure. (**c**) Lifespans of N2 worms exposed to shock waves in S-medium or polyvinyl alcohol. (**d**) Lifespans of N2 worms exposed to shock waves in S-medium or polyvinyl alcohol, followed by a short washing step. (**e**) Lifespans of *daf-16(m26)* worms following shock wave exposure. (**f**) Lifespans of N2 worms at 11 °C following shock wave exposure. (**g**) Lifespans of *daf-2(e1370)* worms at 11 °C following exposure to shock waves. In all panels, vertical dotted lines represent 50% lifespan of control. Arrows and horizontal dotted lines represent the percent survival of each test group at time of the corresponding 50% control survival. N > 500 worms in all experiment groups (the exact numbers are provided in the Methods section).

In order to evaluate *C. elegans* as a potential model for the long term effects of br-mTBI, we exposed wild-type *C. elegans* to shock waves and measured lifespan following exposure. Using a therapeutic shock wave device, shock waves with similar wave properties as primary blast waves were applied as previously described^9^. Exposure to shock waves resulted in a significantly reduced lifespan of both worms raised in liquid cultures (Fig. 1a) and worms raised on NGM-agar plates (Fig. 1b). Furthermore, worms exposed to 500 shock waves exhibited significantly shorter lifespans compared to those exposed to 100 shock waves, particularly in worms raised on NGM-agar plates, indicating a dose-dependent long term effect of shock wave exposure.

It has previously been established that cavitation plays a large role in shock wave effect on nervous tissue^10^. Cavitation has also been hypothesized to be a damaging factor in the induction of br-mTBI^11^. We previously showed that using polyvinyl alcohol (PVA), a medium with low cavitation activity, as a medium for exposing *C. elegans* to shock waves, resulted in significantly reduced immediate effect^9^. In order to investigate the role of cavitation in long term damage following shock wave exposure, we exposed *C. elegans* to shock waves while in PVA. Attenuating cavitation resulted in significantly longer lifespans, although still significantly lower than control-level, despite a potentially toxic effect observed when using PVA without shock waves (Fig. 1c). Any toxic effect of PVA was effectively mitigated using a short wash step prior to putting worms on NGM-agar plates (Fig. 1d).

Knockout *C. elegans* were previously observed with varied lifespans from wild-type N2 *C. elegans*. While *daf-2(e1370)* mutants can live more than two times longer than N2 *C. elegans*, the gene knockout observed in *daf-16(m26)* mutants also plays a role in the aging pathway^12^. In order to investigate the interaction of shock wave exposure and other lifespan shortening factors, we exposed *daf-16* and *daf-2* worms to shock waves. We found that exposure to shock waves induced a significant further reduction in lifespan of *daf-16* worms (Fig. 1e), although the reduction in lifespan was not as pronounced as in N2 *C. elegans* (Fig. 1a). Following exposure to shock waves and maintenance at 11 °C, N2 (Fig. 1f) and *daf-2* worms (Fig. 1g) exhibited significant reduction of lifespan, although lifespan in *daf-2* worms was still longer than that of N2 counterparts at 11 °C.

Wild-type N2 *C. elegans* maintained at 11 °C following shock wave exposure exhibited significantly shorter lifespan compared to control worms at 11 °C (Fig. 1f). The initial damaging effect of shock wave was evident through the first several days and similar to the N2 worms assayed at 20 °C. In the 20 °C assay (Fig. 1a), at the point when 95% of control worms remained alive (t = 8.0 days), 73.2% of worms exposed to 500 shock waves remained alive. At the 95% living mark of worms at 11 °C (t = 8.6 days) a similar amount of 74.0% of worms remained alive. However, long-term disparity between control worms and those exposed to shock waves was less evident. At the 50% survival point of control worms at 20 °C, only 9.7% and 8.2% of worms survived following exposure to 100 and 500 shock waves, respectively. For the worms assayed at 11 °C, those figures were much higher at 35.5% and 26.5%, respectively (Table 1). At the 5% survival point of worms exposed to 500 shock waves at 20 °C (t = 21.5 days), 38.3% of control worms remained alive. For worms maintained at 11 °C, only 10.4% of control worms remained alive when 5% of worms exposed to 500 shock waves remained alive (t = 27.7 days). These data imply that there is an initial death-causing effect of shock wave exposure observed in the first days, as well as a longer term effect on those worms that survive the initial damaging effect. While the initial effects do not appear to be attenuated by the lower temperature, the long-term prognosis of worms exposed to shock waves is much closer to that of control worms. The lack of improved short-term recovery in hypothermia treated worms seems to agree with previous findings of no improvement in the six month mortality rate of traumatic brain injury patients treated with hypothermia^5^. Of note, the six month recovery endpoint used in the aforementioned study correlates to approximately six hours of *C. elegans* recovery. Thus, the findings in the present study of attenuated long-term effects following hypothermia treatment represent findings of a different scope than in previous studies.

**Table 1:** Summary of results.

		Remaining Lifespan at 50% Control			
Assay	50% Lifespan of Control	% of 100 SW	% of 500 SW	% of PVA	% of PVA + 500 SW
Liquid	20.2 days	9.7%	8.2%	—	—
Plates	15.1 days	26.5%	13.4%	—	—
PVA	17.6 days	—	16.0%	34.1%	22.6%
PVA with Wash	16.6 days	—	26.6%	48.7%	33.9%
*daf-16(m26)*	12.2 days	36.9%	33.5%	—	—
11 °C N2	23.4 days	35.5%	26.5%	—	—
11 °C *daf-2(e1370)*	26.7 days	39.1%	16.9%	—	—

## Conclusion

Our results show that *C. elegans* model the long term effects of br-mTBI by showing reduction of lifespan following exposure to shock waves. This effect is reduced in the presence of cavitation, a suspected damage causing component of primary blast waves. Furthermore, there appear to be separate short- and long-term effects of shock wave exposure on *C. elegans*, the latter of which can be attenuated to some extent in reduced temperature settings. This lends credence to usage of therapeutic hypothermia following br-mTBI. Given the utility of *C. elegans*, we believe that the model described in the present study offers a unique opportunity to further understand and investigate long term effects of br-mTBI, especially as they pertain to therapeutic approaches such as therapeutic hypothermia.

## Methods

### Nematodes

All *C. elegans* strains were obtained from the Caenorhabditis Genetics Center (Minneapolis, MN, USA), which is funded by NIH Office of Research Infrastructure Programs (P40 OD010440). The following *C. elegans* strains were used in the present study: Wild type (N2, Bristol), DR26 *daf-16*(*m26*), and CB1370 *daf-2*(*e1370*). From a stock liquid culture, synchronous young adult worms were produced using sodium hypochlorite treatment and sucrose cleaning as previously described^1^. Worms in liquid cultures were allowed to grow in an incubated shaker (NB 205V, N Biotek, Bucheon, South Korea) at 20 °C, while worms raised on plates were kept in an incubator at 20 °C.

### Assays

Seven different assays were performed in the present study. Unless otherwise noted, worms were raised in liquid cultures containing S-medium^13^ and allowed to grow to the L4 stage at 20 °C. At this point, worms were cleaned using sucrose cleaning and prepared for use by concentrating via centrifugation to approximately 200 worms/mL. From this dilution, 310 µL were added to a well of a 96-well plate. When called for, shock waves were applied using a therapeutic shock wave device (Swiss DolorClast; Electro Medical Systems, S. A., Nyon, Switzerland) as previously described^9^. Following shock wave application, worms were transferred using a rapid transfer method outlined previously^9^ to 10 cm NGM-agar plates containing 400 M 5-Fluoro-2′-deoxyuridine (FUdR) in order to prevent future generations of worms to hinder counting^14^. Each group contained at least 500 worms across 10 FUdR NGM-agar plates. Counting commenced on day zero, corresponding with the young adult stage, and occurred every 2 days until no living worms remained. Death was scored as the absence of any movement and failure to move at all after several light pokes with a platinum wire. The following assays were performed in the present study (the numbers in parentheses represent the numbers of worms per individual assay).

#### Assay 1

N2 worms were exposed to 0, 100, or 500 shock waves (0 shock waves: 502 worms; 100 shock waves: 508 worms; 500 shock waves: 507 worms).

#### Assay 2

N2 worms were raised synchronously on 10 cm NGM-agar plates. On day zero (i.e. at the young adult stage), worms were rinsed off of plates with S-medium. Worms were centrifuged to concentrate to 200 worms/mL. Samples were then exposed to 0, 100, or 500 shock waves (0 shock waves: 524 worms, 100 shock waves: 515 worms; 500 shock waves: 518 worms).

#### Assay 3

N2 worms were exposed to either 0 or 500 shock waves in either S-medium or PVA (0 shock waves, S-medium: 511 worms; 500 shock waves, S-medium: 544 worms; 0 shock waves, PVA: 528 worms; 500 shock waves, PVA: 510 worms).

#### Assay 4

N2 worms were exposed to either 0 or 500 shock waves in either S-medium or PVA, including an added washing step following shock wave exposure (0 shock waves, S-medium: 502 worms; 500 shock waves, S-medium: 506 worms; 0 shock waves, PVA: 507 worms; 500 shock waves, PVA: 522 worms).

#### Assay 5

DR26 worms were exposed to 0, 100, or 500 shock waves in S-medium (0 shock waves: 531 worms; 100 shock waves: 583 worms; 500 shock waves: 543 worms).

#### Assay 6

N2 worms were raised at 11 °C, exposed to 0, 100, or 500 shock waves in S-medium, and assayed at 11 °C (0 shock waves: 568 worms; 100 shock waves: 513 worms; 500 shock waves: 537 worms).

#### Assay 7

CB1370 worms were raised at 11 °C, exposed to 0, 100, or 500 shock waves in S-medium, and assayed at 11 °C (0 shock waves: 507 worms; 100 shock waves: 503 worms; 500 shock waves: 510 worms).

### Data Analysis

Data was analyzed with IBM SPSS Statistics (version 23, IBM Corp., Armonk, NY) using the Kaplan Meier Mantel-Cox log rank test. A p value of 0.05 was used as the criteria for significance. GraphPad Prism (Version 5.04) was used to make Fig. 1.

### Data availability

The data that support the findings of this study are available from the corresponding author upon reasonable request.
